# Multimodal Imaging of Diabetic Retinopathy: Insights from Optical Coherence Tomography Angiography and Adaptive Optics

**DOI:** 10.3390/diagnostics15141732

**Published:** 2025-07-08

**Authors:** Andrada-Elena Mirescu, Dan George Deleanu, Sanda Jurja, Alina Popa-Cherecheanu, Florian Balta, Gerhard Garhofer, George Balta, Irina-Elena Cristescu, Ioana Teodora Tofolean

**Affiliations:** 1 “Ovidius” University of Constanta, 900470 Constanta, Romania; 2 “Carol Davila” University of Medicine and Pharmacy, 050747 Bucharest, Romania; 3 County Clinical Emergency Hospital of Constanta, 900591 Constanta, Romania; 4 Department of Ophthalmology, University Emergency Hospital, 050098 Bucharest, Romania; 5 Clinical Emergency Eye Hospital, 030167 Bucharest, Romania; 6 Retina Clinic, 014142 Bucharest, Romania; 7 Department of Clinical Pharmacology, Medical University Vienna, 1090 Vienna, Austria

**Keywords:** diabetic retinopathy, optical coherence tomography angiography, adaptive optics ophthalmoscopy

## Abstract

**Background/Objectives**: To investigate the role of multimodal imaging, specifically optical coherence tomography angiography (OCTA) and adaptive optics (AO), in the diagnosis and monitoring of diabetic retinopathy. **Methods**: Our study represents an observational, cross-sectional analysis including sixty-nine patients from four distinct groups: a control group (17 patients), diabetic patients without diabetic retinopathy (no DR) (14 patients), diabetic patients with non-proliferative diabetic retinopathy (NPDR) (18 patients), and diabetic patients with proliferative diabetic retinopathy (PDR patients). A comprehensive ophthalmological evaluation, along with high-resolution imaging using OCTA and AO, was performed. OCTA images of the superficial capillary plexus, acquired with the OCT Angio Topcon, were analyzed using a custom-developed MATLAB algorithm, while AO retinal vascular images were evaluated with the manufacturer’s software of the Adaptive Optics Retinal Camera rtx1™. **Results**: Our findings demonstrated statistically significant reductions in foveal avascular zone circularity, superficial capillary plexus density, vessel length density, and fractal dimension, correlating with the severity of diabetic retinopathy, particularly in the PDR. Additionally, mean wall thickness and wall-to-lumen ratio were significantly increased in patients with diabetic retinopathy, notably in PDR. **Conclusions**: In conclusion, our findings demonstrate that the combined use of OCTA and AO imaging offers complementary insights into the microvascular alterations associated with diabetic retinopathy progression and severity. These high-resolution modalities together reveal both perfusion deficits and structural vascular changes, underscoring their utility as essential tools for early detection, staging, monitoring, and informed management of DR.

## 1. Introduction

Diabetic retinopathy (DR) is a prevalent microvascular complication of diabetes mellitus and is among the leading causes of preventable blindness worldwide [1,2]. It is also one of the fastest-growing chronic diseases globally, with projections indicating that the number of individuals affected by diabetes mellitus will rise to approximately 642 million by 2040 [3,4]. As diabetes becomes more prevalent, coupled with longer lifespans and a growing elderly population, the burden of vision impairment linked to diabetic retinopathy is likely to escalate considerably in the future [3]. The initial diagnosis of DR is established by performing a clinical fundoscopic examination [3]. Diabetic retinopathy is generally classified into two main forms: non-proliferative diabetic retinopathy (NPDR) and proliferative diabetic retinopathy (PDR), the latter characterized by the formation of new blood vessels (neovascularization) [1]. With the progression of diabetic retinopathy, various complications can arise, such as cotton wool spots, venous beading, and intraretinal microvascular abnormalities (IRMAs) [5,6]. Additionally, as diabetic retinopathy advances into its proliferative stage, retinal neovascularization can form, potentially resulting in severe complications like preretinal bleeding, vitreous hemorrhage, and tractional retinal detachment [6]. Patients affected by DR may also face additional complications, including diabetic macular edema (DME) and diabetic macular ischemia (DMI), further increasing the risk of vision impairment [7,8].

Multiple imaging techniques are increasingly available for the screening, assessment, diagnosis, and management of diabetic retinopathy. At present, dye-based fluorescein angiography remains the gold standard for evaluating the degree of vascular leakage and detecting areas of retinal ischemia [3]. Optical coherence tomography angiography (OCTA) is emerging as a promising, rapid, and non-invasive imaging technique for evaluating microvascular alterations at the capillary level [9,10]. It operates by capturing multiple OCT B-scans at the same retinal location to detect variations in reflectance signals caused by the movement of red blood cells within blood vessels [3]. OCTA enables visualization of the retinal microvasculature at different depths without the need for intravenous dye injections, allowing individual assessment of microvascular changes within the superficial, intermediate, and deep capillary plexuses [5,11]. Essentially, OCTA produces a detailed map of retinal microvasculature derived from variations in optical coherence tomography (OCT) signal intensity caused by blood cell motion [5]. While OCTA technology does not visualize leakage from blood vessels, unlike fluorescein angiography (FA), it offers several benefits over traditional FA imaging [3]. First, OCTA is non-invasive and does not require dye injections, enabling detailed visualization of retinal microvascular changes. This allows OCTA scans to be performed more frequently than fluorescein angiography (FA), making it valuable for patients in need of frequent monitoring [3]. OCTA also allows faster image acquisition compared to fluorescein angiography (FA). Moreover, OCTA captures three-dimensional data with depth-specific resolution, enabling separate visualization and evaluation of different capillary plexuses [3]. Advanced software algorithms can automatically produce images of superficial and deep retinal capillary layers while also permitting user-adjusted segmentation to display additional layers, including intermediate capillary plexuses [3]. This is in contrast with the two-dimensional images obtained using FA, which mainly correspond to the superficial retinal vessels, while deeper capillaries mainly remain unmapped, possibly due to light being scattered at the retina level [3,12,13,14].

Multiple studies have demonstrated that OCTA can be effective in detecting the earliest signs of capillary loss even before the clinical lesions of DR become clinically visible [15,16,17,18]. It can also quantitatively assess the severity of the disease and its associated complications, such as diabetic macular ischemia (DMI) [16,19,20,21,22,23]. There are numerous OCTA metrics that can be used to evaluate disease severity and progression. In diabetic retinopathy (DR), the foveal avascular zone (FAZ) enlarges due to capillary loss in the surrounding vessels [24]. As a result, the most common method for assessment is measuring the FAZ area [3]. Measurements of the foveal avascular zone (FAZ) can offer valuable insights into the status and progression of diabetic retinopathy (DR), diabetic macular edema (DME), and diabetic macular ischemia (DMI) [20]. An enlarged FAZ is a key indicator of DMI severity [25,26,27,28,29] and has been shown to significantly predict DR progression [30]. Some studies even tried to find a connection between FAZ size and the decline in visual acuity [25,28,31]. Research indicates that FAZ size does not always strongly correlate with best-corrected visual acuity (BCVA), as vision loss may lag behind capillary dropout [5]. A critical level of vascular loss must be reached before noticeable impairment occurs, making it difficult to define a precise FAZ size threshold linked to visual decline [5]. As a substantial variation of the FAZ area has been reported in normal eyes (from 0.071 mm^2^ to 0.527 mm^2^) [32,33], evaluating FAZ circularity may provide a more accurate reflection of disease-related microvascular changes than FAZ area alone, as the FAZ typically appears round or slightly elliptical in healthy eyes [33]. In patients with DR, the FAZ tends to lose its circular shape and become more irregular or tortuous [34]. This occurs as capillary dropout at different sites expands, leading to a decrease in circularity. Circularity serves as a mathematical measure of how closely the FAZ resembles a perfect circle [33]. A circularity value of 1.0 represents a perfect circle, while lower values indicate increasing deviation from a circular shape [33]. Circularity is mathematically defined by the formula: Circularity = 4π × area/perimeter^2^. The FAZ area (mm^2^) is determined by counting the total number of pixels within the region [2]. Its contour is then precisely outlined, and the perimeter is calculated by measuring the pixel-to-pixel distance in scale [2]. An increase in FAZ area was also associated with a shorter axial length [2].

Vessel density (VD) is typically described as the ratio of the blood vessel area to the total measured area in a binarized image, serving as an indicator of retinal microvascular perfusion [3,35]. Vessel length density (VLD), also referred to as skeleton density (SD), is considered an alternative metric to vessel density (VD), as it measures vascular density by determining the presence of vessels per unit area without factoring in their diameters [36]. The skeletonization process reduces each vessel to a single-pixel width, enabling the calculation of total vessel length [5,36]. Because all vessels, regardless of size, are represented as single-pixel lines, both large and small capillaries contribute equally to VLD measurements [3,37]. This makes VLD particularly sensitive to detecting perfusion changes at the capillary level [37]. In contrast, non-skeletonized approaches take vessel size into account, offering a more comprehensive and physiologically relevant depiction of the overall vascular network [5,38].

Fractal dimension (FD) is a measure of the complexity of vascular branching patterns. It is determined using the box-counting method, which involves overlaying a grid of varying square sizes onto a skeletonized version of the vasculature and counting the number of occupied boxes. This process quantifies the structural intricacy of the vascular network. To calculate FD, the binarized image is first skeletonized, followed by the application of the box-counting method to assess its fractal properties [2,3]. A higher FD value indicates a more complex and dense vascular structure, while a lower FD suggests a simpler or more degraded network [2].

Studies conclude that diabetic retinopathy severity is correlated with an enlarged FAZ area, decreased FAZ circularity, lower VD, and decreased FD [2,3].

Historically, the evaluation of retinal tissue at a cellular scale depended predominantly on histological approaches, as conventional imaging modalities lacked adequate resolution due to inherent optical aberrations within the human eye [39]. The emergence of adaptive optics (AO) technology addressed this limitation by correcting wavefront distortions, substantially improving optical resolution to approximately 2 µm [39]. Consequently, AO has enabled high-resolution, non-invasive, in vivo imaging of retinal microstructures, including individual photoreceptors and microvasculature [39,40].

Adaptive optics (AO) imaging has become an essential tool in ophthalmology, particularly in assessing retinal vasculature in diabetic patients [41]. This imaging modality allows precise measurement of vascular morphology by quantifying parameters such as total vessel diameter (TVD), lumen diameter (LD), and mean wall thickness (WT) [42]. Additionally, the wall-to-lumen ratio (WLR), calculated as the ratio of wall thickness to lumen diameter, provides valuable insights into vascular integrity [42]. Since the WLR typically follows a predictable pattern for a given vessel size, deviations from this normative relationship can potentially serve as biomarkers, indicating early vascular changes associated with diabetic retinal disease [43].

Adaptive optics fundus camera (AO-FC) imaging has revealed substantial morphological alterations in retinal vessels among patients with diabetes mellitus [41,44]. Notably, arterial wall thickness was increased in patients diagnosed with diabetic retinopathy (DR) compared to healthy controls [41]. Studies that assessed retinal vascular parameters in individuals with type 2 diabetes revealed a considerably elevated wall-to-lumen ratio (WLR) in patients with proliferative diabetic retinopathy (PDR) relative to less severe disease stages [41]. Furthermore, others identified a positive association between the WLR and various clinical factors, including DR severity, disease duration, elevated systolic blood pressure, and systemic hypertension [44]. These findings suggest that WLR could serve as a reliable indicator of vascular impairment and disease progression in diabetic retinopathy [41,44].

## 2. Materials and Methods

The current study was a cross-sectional observational study conducted on 69 patients from four distinct groups, all evaluated at a single time point in our ophthalmology “Retina” Clinic: a control group (17 patients), diabetic patients without diabetic retinopathy (no DR) (14 patients), diabetic patients with non-proliferative diabetic retinopathy (NPDR) (18 patients), and diabetic patients with proliferative diabetic retinopathy (PDR) (20 patients). All participants were examined at the Retina Clinic in Bucharest, Romania, and provided written informed consent. The study was conducted in accordance with the Declaration of Helsinki and approved by the Ethics Committee of Ponderas Academic Hospital, Bucharest, Romania (No. 331/18 December 2020), and by the Ethics Committee of the “Ovidius” University of Constanţa, Romania (No. UOC 17057/11 November 2022).

### 2.1. Study Participants

To be eligible for inclusion in the study, participants had to be adults (≥18 years old) and capable of understanding the ophthalmological investigations detailed in the study protocol. Inclusion criteria for the control group were age ≥ 18 years, Caucasian ethnicity, absence of significant systemic conditions (including diabetes mellitus), no prescription medication use, best-corrected visual acuity (BCVA) better than 0.3 on the Snellen chart, central fixation with stable fixation during image acquisition, clear ocular media, and refractive error less than 3.0 diopters (spherical) or 2.5 diopters (cylindrical). Common inclusion criteria for the diabetic groups were age > 18 years, Caucasian ethnicity, diagnosis with type I or type II diabetes mellitus at the time of inclusion in our study, best-corrected visual acuity (BCVA) better than 0.3 on the Snellen chart, central fixation with stable fixation during image acquisition, ocular media sufficiently clear to permit high-quality imaging, refractive error less than 3.0 diopters (spherical) or 2.5 diopters (cylindrical), and no ophthalmologic interventions within the past 3 months. The diabetic patients were further categorized into three subgroups: patients with diabetes mellitus without retinopathy (no DR group), those with non-proliferative diabetic retinopathy (NPDR group), and those with proliferative diabetic retinopathy (PDR group).

### 2.2. Ophthalmic Examination

All participants underwent a comprehensive ophthalmological evaluation at the time of inclusion, which included best-corrected visual acuity assessment, refraction, intraocular pressure measurement, and slit-lamp biomicroscopy of both anterior and posterior segments. To enhance posterior segment visualization and optimize image acquisition with OCTA and adaptive optics, pharmacologic mydriasis was induced using 10% phenylephrine and 1% tropicamide. Imaging was performed using a swept-source optical coherence tomography device (DRI OCT Triton, Topcon Inc., Tokyo, Japan), along with an optical coherence tomography angiography system (OCT Angio, Topcon) and the Adaptive Optics Retinal Camera rtx1™ (Imagine Eyes, Orsay, France). Axial length was measured using the IOL Master 700 (Carl Zeiss Meditec, Jena, Germany).

### 2.3. Image Processing

#### 2.3.1. Optical Coherence Tomography Angiography

Regarding OCTA analysis, image acquisition as a 4.5 × 4.5 mm (320 × 320 pixels) en face image was followed by automated processing using IMAGEnet 6 software (Topcon Inc.). The software also performed automated retinal layer segmentation, defining the superficial retinal layer, considered to represent the superficial capillary plexus (SCP), as the region from 2.6 μm below the internal limiting membrane (ILM) to 15.6 μm below the interface between the inner plexiform layer (IPL) and the inner nuclear layer (INL).

OCTA images of the superficial capillary plexus were imported into a custom-developed MATLAB algorithm (MATLAB R2024b, The MathWorks Inc., Natick, MA, USA) for further analysis.

**Figure 1:**
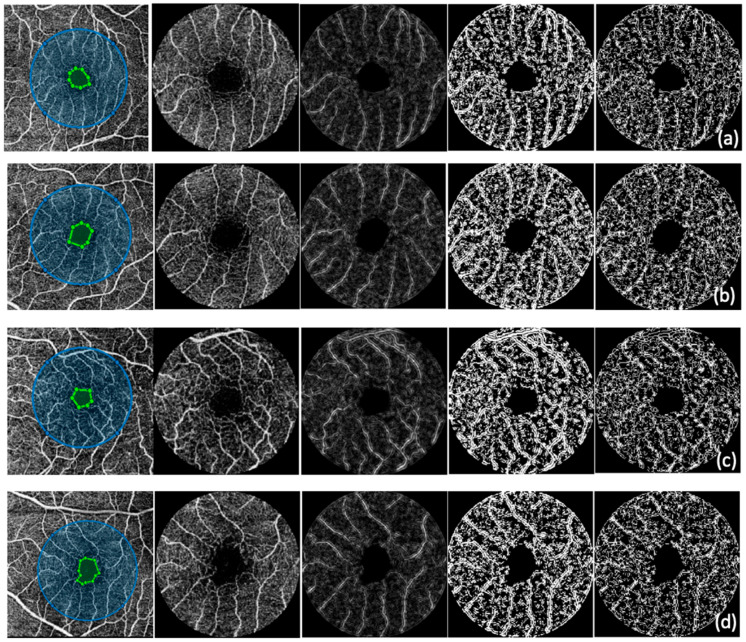
OCTA image of (**a**) a healthy volunteer, (**b**) a no DR patient, (**c**) an NPDR patient, and (**d**) a PDR patient; custom-developed MATLAB algorithm—for left to right, the first picture represents a manually drawn FAZ (area marked by green dots) and a 3 mm diameter circle (blue circle), the second picture represents the cropped image with FAZ excluded, the third image represents the 2D Frangi vesselness filter, the fourth image represents the binarized image using the Phansalkar local thresholding method, and the fifth image represents the skeletonized image.

The workflow consisted of the following steps for each of the SCP images: (1) The foveal avascular zone (FAZ) was drawn manually, and its centroid was used to define a 3 mm diameter circle, which was then cropped for further analysis. (2) The FAZ area, perimeter, and circularity index were automatically computed based on the FAZ mask within the cropped region, and further FAZ area and perimeter values were adjusted for axial length. (3) The cropped image was then converted to grayscale, and the drawn FAZ area was excluded from further analysis by masking it out. (4) The vascular structures were enhanced using a 2D Frangi vesselness filter [45], and the output vesselness image was inverted to highlight vascular structures. (5) The inverted vesselness image was binarized using the Phansalkar local thresholding method [46]. (6) Vessel density was calculated on the binarized image as the ratio of binarized vessel pixels to the total area. (7) The binarized image was then skeletonized. (8) Vessel length density was computed as the ratio of skeleton length to the total area. (9) The fractal dimension was estimated using a box-counting method to quantify vascular complexity (Figure 1).

#### 2.3.2. Adaptive Optics

**Figure 2:**
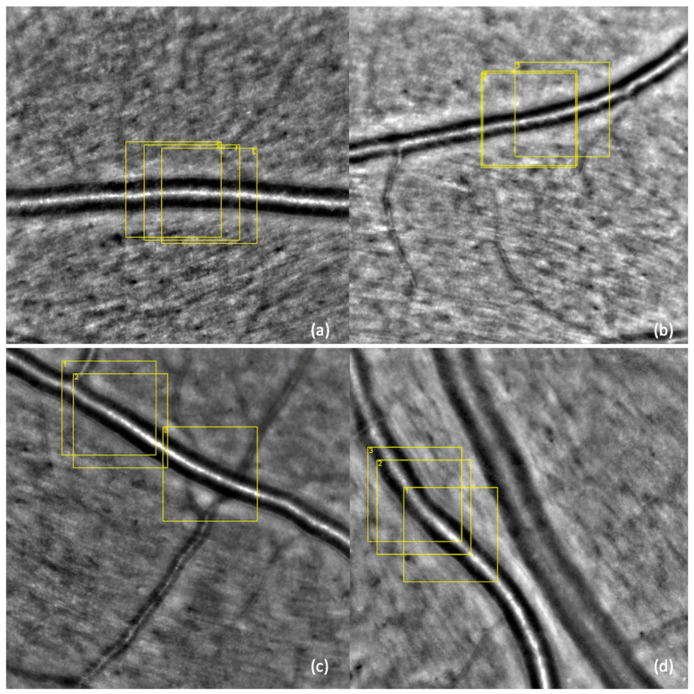
Adaptive optics ophthalmoscopy image—image of the retinal arteriole of (**a**) a healthy volunteer, (**b**) a no DR patient, (**c**) an NPDR patient, and (**d**) a PDR patient; the yellow boxes represent the selected regions of interest (ROIs), each measuring 100 microns, as indicated by the scale bar and three consecutive measurements were performed, as indicated by the numbers next to each yellow box.

Adaptive optics images were analyzed using the manufacturer’s software (AOdetect Artery, Imagine Eyes, France), which automatically generates the following vascular parameters for the selected regions of interest (ROIs) each measuring 100 microns, as indicated by the scale bar: total vessel diameter (TVD), lumen diameter (LD), mean wall thickness (WT), wall-to-lumen ratio (WLR), and cross-sectional area of the vascular wall (WCSA). Three distinct ROIs were analyzed for each image, and the mean of the three measurements was used for each parameter. All values were adjusted for axial length. (Figure 2). A total of sixteen patients were excluded from data collection due to lack of cooperation or time constraints. Specifically, five patients were excluded from the control group, one from the no DR group, three from the NPDR group, and seven from the PDR group.

### 2.4. Statistical Analysis

The statistical analyses were performed using GraphPad Prism software (version 10.4.2). The Shapiro–Wilk test was used to assess the normality of continuous variable distributions. Statistical differences between the four groups for each parameter (including group characteristics, OCTA, and AO metrics) were calculated using the Kruskal–Wallis test (results are presented as H statistics with corresponding *p*-values) when the data were not normally distributed or one-way ANOVA (results are presented as F statistics with corresponding *p*-values) when data from all groups followed a normal distribution. When statistically significant differences were found (*p* < 0.05), multiple post hoc comparisons between groups were performed using Dunn’s test (following Kruskal–Wallis) or Tukey’s multiple comparisons test (following ANOVA), with results expressed as adjusted *p*-values (*p* adj), as appropriate.

## 3. Results

Sixty-nine patients were included in our study: 17 from healthy volunteers (11 male and 6 female) and 52 from diabetic patients (30 male and 22 female), including 12 patients with type I diabetes mellitus and 40 with type II diabetes mellitus, of whom 26 were insulin-dependent at the time of inclusion (Table 1). Of the 38 patients with NPDR or PDR, 17 had associated maculopathy at the time of inclusion, 14 had previously undergone panretinal photocoagulation, 19 had received intravitreal anti-vascular endothelial growth factor (anti-VEGF) injections, and 5 had undergone pars plana vitrectomy. All procedures had been performed more than three months prior to inclusion in the study.

**Figure 3:**
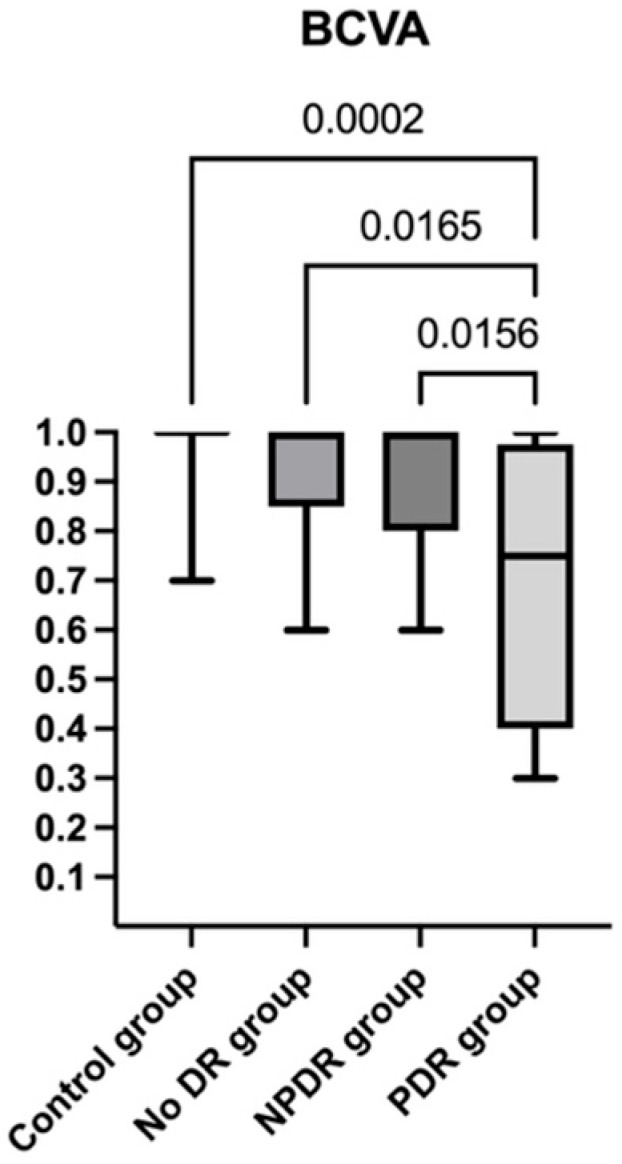
Best corrected visual acuity (BCVA) distribution and data analysis among study groups; post hoc analyses (Dunn’s test) and the statistically significant adjusted *p*-values between groups: control group and PDR group (*p* adj = 0.0002), no DR group and PDR group (*p* adj = 0.016), and NPDR group and PDR group (*p* adj = 0.015).

There was no significant difference in age between groups (F(3, 65) = 0.495, *p* = 0.686). However, a significant difference was found in BCVA (H(3) = 19.89, *p* = 0.0002); post hoc Dunn’s test showed significant differences between the control group and the PDR group (*p* adj = 0.0002), the no DR group and the PDR group (*p* adj = 0.016), and the NPDR group and the PDR group (*p* adj = 0.015). BCVA significantly decreased as diabetic retinopathy progressed, from a mean ± standard deviation of 0.976 ± 0.007 in the control group to 0.921 ± 0.142 in the no DR group, 0.916 ± 0.133 in the NPDR group, and 0.685 ± 0.271 in the PDR group (Figure 3).

A wide range of OCTA and AO parameters were considered in the statistical analyses (Table 2).

**Figure 4:**
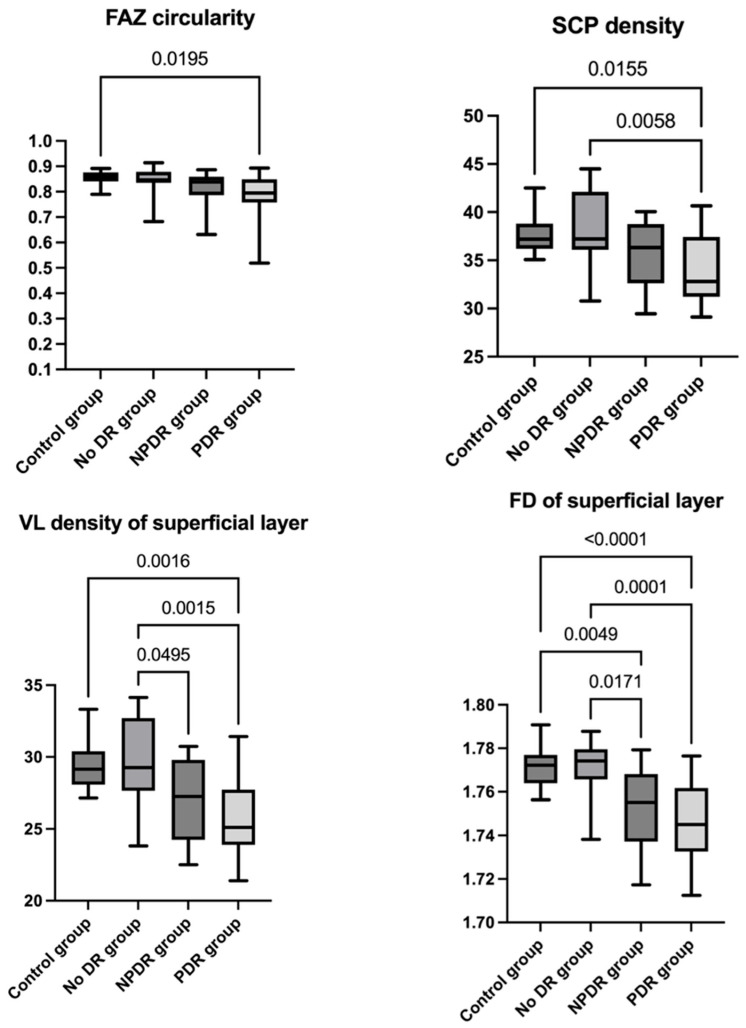
FAZ circularity, SCP density, VL density of superficial layer, FD of superficial layer distribution among the study groups and data analyses; FAZ = foveal avascular zone, SCP = superficial capillary plexus, VL = vessel length, FD = fractal dimension; post hoc analyses (Dunn’s test) and the statistically significant adjusted *p*-values between groups for FAZ circularity: control group and PDR group (*p* adj = 0.019); post hoc analyses (Tukey’s test) and the statistically significant adjusted *p*-values between groups for SCP density: control group and PDR group (*p* adj = 0.015), no DR group and PDR group (*p* adj = 0.005), for VL density of superficial layer: control group and PDR group (*p* adj = 0.001), no DR group and PDR group (*p* adj = 0.001), no DR group and NPDR group (*p* adj = 0.049), and for FD of superficial layer: control group and PDR group (*p* adj < 0.0001), no DR group and PDR group (*p* adj = 0.0001), control group and NPDR group (*p* adj = 0.004), no DR group and NPDR group (*p* adj = 0.017).

Regarding OCTA parameter analysis, no significant differences were found in FAZ area (F(3, 65) = 0.644, *p* = 0.589) or FAZ perimeter (F(3, 65) = 1.225, *p* = 0.307) between groups. However, FAZ circularity showed a significant difference (H(3) = 10.71, *p* = 0.013); post hoc Dunn’s test revealed a significantly reduced FAZ circularity in the PDR group compared to the control group (*p* adj = 0.019). Superficial capillary plexus density also differed significantly across groups (F(3, 65) = 5.249, *p* = 0.002); Tukey’s multiple comparisons test indicated a significantly lower SCP density in the PDR group compared to the control group (*p* adj = 0.015) and the no DR group (*p* adj = 0.005). Vessel length density of the superficial layer showed a significant difference between groups (F(3, 65) = 7.542, *p* = 0.0002); post hoc Tukey’s test revealed a significantly lower VL density in the PDR group compared to both the control group (*p* adj = 0.001) and the no DR group (*p* adj = 0.001). Additionally, the NPDR group showed significantly lower values compared to the no DR group (*p* adj = 0.049). Fractal dimension of the superficial layer was also significantly different between groups (F(3, 65) = 11.87, *p* < 0.0001); post hoc Tukey’s test showed a significantly lower FD in the PDR group compared to both the control group (*p* adj < 0.0001) and the no DR group (*p* adj = 0.0001). Additionally, the NPDR group demonstrated a significantly reduced FD compared to the control group (*p* adj = 0.004) and the no DR group (*p* adj = 0.017) (Figure 4) (Table 3).

**Figure 5:**
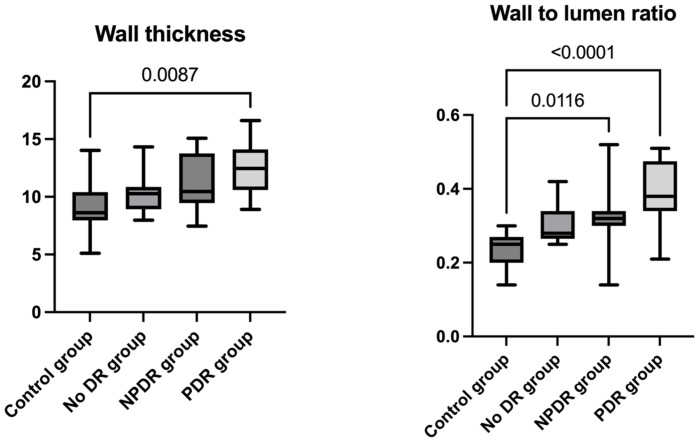
WT, WLR distribution among the study groups, and data analyses; WT = mean wall thickness, WLR = wall-to-lumen ratio; post hoc analyses (Dunn’s test) and the statistically significant adjusted *p*-values between groups for WT: control group and PDR group (*p* adj = 0.008), and for WLR: control group and PDR group (*p* adj < 0.0001), and control group and NPDR group (*p* adj = 0.011).

There were no significant differences between the groups regarding total vessel diameter (H(3) = 1.082, *p* = 0.781), lumen diameter (H(3) = 6.431, *p* = 0.092), or cross-sectional area of the vascular wall (H(3) = 2.132, *p* = 0.545). However, significant differences were found for mean wall thickness (H(3) = 10.95, *p* = 0.012) and wall-to-lumen ratio (H(3) = 21.98, *p* < 0.0001). Post hoc Dunn’s test revealed a significantly increased wall thickness in the PDR group compared to the control group (*p* adj = 0.008) and significantly higher WLR in the PDR group compared to the control group (*p* adj < 0.0001) as well as in the NPDR group compared to the control group (*p* adj = 0.011) (Figure 5) (Table 3).

**Figure 6:**
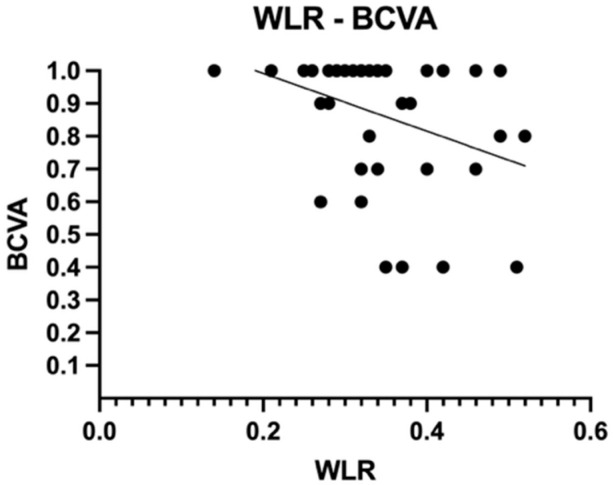
Correlation between BCVA and WLR in diabetic patients; BCVA = best corrected visual acuity, WLR = wall-to-lumen ratio.

To assess the correlation between anatomical changes and visual function, we applied Spearman’s rank-order correlation to evaluate the relationship between BCVA and all previously mentioned OCTA and AO parameters in diabetic patients. A statistically significant, moderate negative correlation was found between BCVA and WLR (rs (41) = −0.400, *p* = 0.009) (Figure 6).

## 4. Discussion

Diabetic retinopathy is one of the leading causes of preventable blindness, and high-resolution imaging techniques are essential for its early detection, monitoring, and management [1,2]. OCTA and AO are non-invasive imaging techniques used in the evaluation of diabetic retinopathy, offering detailed insights into the retinal microvasculature. OCTA enables the early detection of capillary dropout and allows for the quantification of FAZ morphology, vessel density alterations, and fractal complexity [3]. AO enhances image resolution, enabling the visualization of fine microvascular structures and the measurement of key vascular parameters, most notably the wall-to-lumen ratio [42]. Together, these techniques offer complementary information on both structural vascular alterations and perfusion status, facilitating early detection, monitoring, and prevention of diabetic retinopathy progression.

The specialty literature shows that diabetic retinopathy severity correlates with several OCTA changes, including FAZ enlargement, reduced circularity, decreased vessel density and vessel length density, and lower fractal dimension [2,3]. In agreement with the literature, our results demonstrated statistically significant reductions in FAZ circularity, superficial capillary plexus density, vessel length density, and fractal dimension, correlating with the severity of diabetic retinopathy, particularly in the PDR group compared to controls or the no DR group. Although the FAZ area and perimeter showed no significant differences between groups, the marked decline in circularity and microvascular metrics underscores progressive capillary loss and structural impairment associated with DR severity.

Literature further highlights that adaptive optics imaging detects early retinal vascular alterations in diabetic retinopathy, with increased wall thickness and elevated wall-to-lumen ratio emerging as reliable markers of structural microvascular damage and disease progression [3]. In agreement with previous reports, our results showed a statistically significant increase in both wall thickness and wall-to-lumen ratio in patients with diabetic retinopathy, particularly in the PDR group. While no significant differences were observed for vessel diameter, lumen diameter, or wall cross-sectional area, the elevated wall-to-lumen ratio and wall thickness reflect early structural vascular changes, reinforcing the literature findings that highlight these parameters as sensitive markers of microvascular damage in DR.

Our study benefits from a multimodal imaging approach, combining OCTA and AO to provide a more comprehensive evaluation of microvascular changes in diabetic patients, both with and without diabetic retinopathy. While previous studies have typically focused on either OCTA or AO in isolation, our work integrates both modalities, allowing simultaneous assessment and quantification of multiple parameters. This combined approach enables the characterization of both functional alterations, such as vessel density, vessel length density, fractal dimension, and structural changes, including vascular wall remodeling. By capturing these complementary aspects, our study offers a deeper understanding of the microvascular alterations associated with diabetic retinopathy.

One notable limitation of our study is the inclusion of some patients in the non-proliferative and proliferative diabetic retinopathy groups who had a history of prior treatments, such as panretinal photocoagulation, intravitreal anti-vascular endothelial growth factor injections, or pars plana vitrectomy. Although all treatments were administered at least three months prior to inclusion in order to minimize short-term effects, we acknowledge that these interventions may still exert lasting influences on retinal vascular parameters. As our clinic serves as a national referral center for advanced retinal pathology, treatment-naive patients with severe diabetic retinopathy are infrequently encountered. Consequently, the differences observed between groups may partially reflect the cumulative impact of both disease severity and prior therapeutic interventions. To address this, we included only patients whose last treatment occurred at least three months before enrollment.

Evidence from the literature indicates that a study has shown that anti–vascular endothelial growth factor (anti-VEGF) therapy generally does not lead to reperfusion of small retinal vessels in areas of nonperfusion, even though neovascularization regressed and diabetic retinopathy severity scale scores may improve [47]. However, a more recent study suggests that intensive treatment with anti-VEGF may reduce areas of nonperfusion in patients with diabetic retinopathy who do not have diabetic macular edema, indicating that significant vascular leakage was strongly linked to retinal reperfusion [48].

Additional evidence from the literature confirms that intravitreal anti-VEGF therapy administered over a 3- to 9-month period is associated with a reduction in vessel diameter and increased circularity of the foveal avascular zone within the superficial retinal vasculature, indicating potential vascular remodeling. These vascular changes were most prominent in patients who experienced improvements in best corrected visual acuity and central subfield thickness, suggesting that OCTA-derived metrics may serve as surrogate markers of treatment responsiveness [49].

It is important to note that several patients in the non-proliferative and proliferative diabetic retinopathy groups had received anti–vascular endothelial growth factor therapy prior to imaging. As shown in this study, anti-VEGF treatment can induce partial restoration of retinal microvascular architecture. Therefore, vascular parameters measured after treatment may underestimate the actual extent of anatomical damage that was present before therapy. The difference in vessel density measurements obtained after anti-VEGF therapy may underestimate the initial extent of capillary dropout, as treatment can improve capillary visibility and, in certain cases, induce partial reperfusion, particularly in patients without diabetic macular edema. Likewise, vessel diameter between diabetic eyes and healthy controls may be underestimated when assessed after anti-VEGF therapy, as the treatment induces vasoconstriction and partially normalizes prior pathological vascular permeability, thereby masking the true baseline severity. And last, but not least, FAZ circularity may be underestimated, since therapy can partially restore its symmetry. If statistically significant differences are observed between these patients and healthy individuals, it is reasonable to assume that the initial differences prior to treatment were even more pronounced.

Panretinal photocoagulation (PRP) has been shown to influence retinal microvasculature as assessed by OCTA. Several studies demonstrate significant increases in macular vessel density and reductions in foveal avascular zone area following PRP. Abdelhalim et al. reported that both superficial and deep capillary plexus VD increased significantly at 1 month and 6 months post-treatment, accompanied by a notable decrease in FAZ area [50]. Similarly, Chatziralli et al. found increased superficial VD and a more circular FAZ over a 12-month follow-up [51]. The suggested mechanisms behind the improved OCTA metrics after PRP are an overall redistribution of blood flow from the occluded peripheral capillaries to the posterior pole as well as an increase in choroidal flow in the subfoveal region [52,53,54].

In contrast, other investigations did not observe meaningful OCTA changes after PRP. Lorusso et al. found no significant change in OCTA parameters after peripheral laser treatment at both 1-month and 6-month follow-ups for 18 eyes [55].

Therefore, regarding our study, vascular parameters assessed after PRP may either underestimate the true extent of anatomical damage that existed prior to treatment or appear unchanged, despite underlying structural alterations.

Pars plana vitrectomy (PPV) is indicated in PDR for complications like non-clearing vitreous hemorrhage or tractional retinal detachment. Surgical removal of the vitreous (often combined with membrane peeling and endolaser PRP during surgery) can relieve retinal traction, remove hemorrhage and fibrovascular tissue, and improve diffusion of oxygen and cytokines within the eye [56]. These effects might translate into changes in retinal microvasculature that are detectable on OCTA.

Some studies suggest that vitrectomy can enhance macular perfusion and vessel density in PDR patients, especially after traction is relieved. Petrou et al. performed OCTA before and 3 months after PPV and found a significant reduction in FAZ size after surgery [57]. Despite the fact that vitrectomy in these cases included variable clinical scenarios, the reduction in the FAZ area was clear even in the cases where the macular status was not influenced directly by the vitrectomy, as in macula-on retinal detachment and vitreous hemorrhage [57].

PPV may lead to localized improvements in retinal perfusion, especially when traction is relieved, but it does not consistently restore lost capillary networks. Any observed OCTA improvements are likely due to mechanical relief of obstruction rather than reversal of diabetic microangiopathy [58].

Another potential limitation of our study is the presence of selection bias in the adaptive optics analysis. Several patients, mainly from the proliferative diabetic retinopathy group, were excluded from AO imaging due to poor fixation, blinking artifacts, or insufficient image quality, which are technical limitations inherent to the acquisition process. These exclusions were based solely on image acquisition constraints and not on clinical status or disease severity. Nevertheless, this may have led to an underrepresentation of patients with more advanced disease in the AO subgroup, potentially underestimating the extent of vascular abnormalities in the PDR group. Despite this, the PDR group remains sufficiently represented for meaningful comparison, and the imaging protocol was applied consistently across all study groups. Importantly, the statistical methods used were designed to normalize and adjust for sample variability across groups, ensuring valid comparisons based on the remaining data.

Adaptive optics imaging is still rarely available in routine clinical settings and requires expensive equipment. Both AO and OCTA involve costs, require patient cooperation, and depend on stable central fixation to ensure optimal image quality, which may pose practical limitations in routine clinical practice. Despite these constraints, the combined use of AO and OCTA may contribute to the development of novel diagnostic standards for diabetic retinopathy by offering a comprehensive microvascular characterization. The complementary insights provided by these technologies could enhance the detection and monitoring of disease progression, and future efforts should explore their potential integration into screening or follow-up protocols for DR.

## 5. Conclusions

To conclude, our results demonstrate that the combination of OCTA and AO imaging provides complementary insights into the microvascular alterations associated with diabetic retinopathy progression and severity. Together, these high-resolution imaging techniques highlight both perfusion deficits and structural vessel changes, supporting their value as essential tools for early detection, staging, monitoring, and appropriate management of DR.

## Figures and Tables

**Table 1:** Characteristics of the study groups (expressed as mean ± standard deviation): DR = diabetic retinopathy, NPDR = non-proliferative diabetic retinopathy, PDR = proliferative diabetic retinopathy.

	Control Group	No DR Group	NPDR Group	PDR Group
Number of patients (eyes)	17	14	18	20
Sex (male/female)	11/6	5/9	10/8	15/5
Age (years)	52.29 ± 13.13	54.07 ± 21.36	50.27 ± 12.31	56 ± 13.33
Diabetes type (type I/type II)	-	4/10	3/15	5/15
Insulin dependence (independent/dependent)	-	4/10	11/7	11/9
Diabetes duration (years)	-	12.11 ± 6.81	15.55 ± 6.80	15.55 ± 9.25
Eye laterality (right/left)	7/10	6/8	11/7	10/10
Axial length (mm)	23.68 ± 1.00	23.89 ± 0.79	23.34 ± 0.38	23.42 ± 0.74

**Table 2:** Vascular parameters measured using both OCTA and AO (expressed as mean ± standard deviation); DR = diabetic retinopathy, NPDR = non-proliferative diabetic retinopathy, PDR = proliferative diabetic retinopathy, OCTA parameters: FAZ = foveal avascular zone, SCP = superficial capillary plexus, VL = vessel length, FD = fractal dimension; AO parameters: TVD = total vessel diameter, LD = lumen diameter, WT = mean wall thickness, WLR = wall-to-lumen ratio, and WCSA = cross-sectional area of the vascular wall.

	Control Group	No DR Group	NPDR Group	PDR Group
FAZ area of superficial layer (mm^2^)	0.226 ± 0.100	0.214 ± 0.060	0.248 ± 0.084	0.248 ± 0.087
FAZ perimeter of superficial layer (mm)	1.774 ± 0.446	1.772 ± 0.293	1.933 ± 0.354	1.958 ± 0.378
FAZ circularity index of superficial layer	0.853 ± 0.028	0.845 ± 0.054	0.816 ± 0.062	0.792 ± 0.081
SCP density (%)	37.693 ± 1.950	38.274 ± 3.799	35.685 ± 3.455	34.338 ± 3.670
VL density of superficial layer (%)	29.385 ± 1.711	29.586 ± 3.020	27.049 ± 2.837	25.971 ± 2.992
FD superficial layer	1.771 ± 0.009	1.770 ± 0.013	1.753 ± 0.018	1.745 ± 0.018
TVD (μm)	100.341 ± 20.234	89.173 ± 14.913	96.458 ± 26.842	88.237 ± 23.184
LD (μm)	81.085 ± 16.323	68.542 ± 12.510	73.820 ± 25.796	60.787 ± 17.906
WT (μm)	9.670 ± 2.676	10.323 ± 1.737	11.042 ± 2.256	12.08 ± 2.610
WLR	0.237 ± 0.048	0.305 ± 0.054	0.320 ± 0.080	0.390 ± 0.090
WCSA (μm^2^)	2842.17 ± 1285.05	2606.04 ± 839.31	3070.66 ± 1199.36	3002.85 ± 1316.49

**Table 3:** Post hoc analyses (Dunn’s test following Kruskal–Wallis or Tukey’s test following ANOVA) and the adjusted *p*-values from intergroup comparisons of vascular parameters obtained using both OCTA and AO; DR = diabetic retinopathy, NPDR = non-proliferative diabetic retinopathy, PDR = proliferative diabetic retinopathy, OCTA parameters: FAZ = foveal avascular zone, SCP = superficial capillary plexus, VL = vessel length, FD = fractal dimension; AO parameters: TVD = total vessel diameter, LD = lumen diameter, WT = mean wall thickness, WLR = wall-to-lumen ratio, WCSA = cross-sectional area of the vascular wall; bold formatting was used to highlight the statistically significant adjusted *p*-values below.

	Control Group and No DR Group	Control Group and NPDR Group	Control Group and PDR Groups	No DR Group and NPDR Group	No DR Group and PDR Group	NPDR Group and PDR Group
FAZ area of superficial layer (mm^2^) (Tukey’s test)	*p* adj = 0.977	*p* adj = 0.870	*p* adj = 0.867	*p* adj = 0.670	*p* adj = 0.661	*p* adj > 0.999
FAZ perimeter of superficial layer (mm) (Tukey’s test)	*p* adj > 0.999	*p* adj = 0.593	*p* adj = 0.450	*p* adj = 0.627	*p* adj = 0.491	*p* adj = 0.997
FAZ circularity index of superficial layer (Dunn’s test)	*p* adj > 0.999	*p* adj = 0.385	***p* adj = 0.019**	*p* adj > 0.999	*p* adj = 0.104	*p* adj > 0.999
SCP density (%) (Tukey’s test)	*p* adj = 0.961	*p* adj = 0.282	***p* adj = 0.015**	*p* adj = 0.133	***p* adj = 0.005**	*p* adj = 0.593
VL density of superficial layer (%) (Tukey’s test)	*p* adj = 0.996	*p* adj = 0.059	***p* adj = 0.001**	***p* adj = 0.049**	***p* adj = 0.001**	*p* adj = 0.609
FD superficial layer (Tukey’s test)	*p* adj = 0.994	***p* adj = 0.004**	***p* adj < 0.0001**	***p* adj = 0.017**	***p* adj = 0.0001**	*p* adj = 0.432
TVD (μm) (Dunn’s test)	*p* adj > 0.999	*p* adj > 0.999	*p* adj > 0.999	*p* adj > 0.999	*p* adj > 0.999	*p* adj > 0.999
LD (μm) (Dunn’s test)	*p* adj = 0.756	*p* adj = 0.967	*p* adj = 0.069	*p* adj > 0.999	*p* adj > 0.999	*p* adj > 0.999
WT (μm) (Dunn’s test)	*p* adj > 0.999	*p* adj = 0.328	***p* adj = 0.008**	*p* adj > 0.999	*p* adj = 0.169	*p* adj = 0.917
WLR (Dunn’s test)	*p* adj = 0.190	***p* adj = 0.011**	***p* adj < 0.0001**	*p* adj > 0.999	*p* adj = 0.064	*p* adj = 0.514
WCSA (μm^2^) (Dunn’s test)	*p* adj > 0.999	*p* adj > 0.999	*p* adj > 0.999	*p* adj > 0.999	*p* adj > 0.999	*p* adj > 0.999

## Data Availability

The original contributions presented in the study are included in the article; further inquiries can be directed to the corresponding authors.

## Abbreviations

The following abbreviations are used in this manuscript:

DR	diabetic retinopathy
NPDR	non-proliferative diabetic retinopathy
PDR	proliferative diabetic retinopathy
IRMAs	intraretinal microvascular abnormalities
DME	diabetic macular edema
DMI	diabetic macular ischemia
OCT	optical coherence tomography
OCTA	optical coherence tomography angiography
FA	fluorescein angiography
FAZ	foveal avascular zone
BCVA	best-corrected visual acuity
VD	vessel density
VLD	vessel length density
FD	fractal dimension
AO	adaptive optics
TVD	total vessel diameter
LD	lumen diameter
WT	mean wall thickness
WLR	wall-to-lumen ratio
WCSA	cross-sectional area of the vascular wall
AO-FC	adaptive optics fundus camera
SCP	superficial capillary plexus
ILM	internal limiting membrane
IPL	inner plexiform layer
INL	inner nuclear layer
ROIs	regions of interest
anti-VEGF	anti-vascular endothelial growth factor
PRP	panretinal photocoagulation
PPV	pars plana vitrectomy

## References

[1] Wong T.Y., Cheung C.M., Larsen M., Sharma S., Simó R. (2016). Diabetic retinopathy. Nat. Rev. Dis. Primers.

[2] Tang F.Y., Ng D.S., Lam A., Luk F., Wong R., Chan C., Mohamed S., Fong A., Lok J., Tso T. (2017). Determinants of Quantitative Optical Coherence Tomography Angiography Metrics in Patients with Diabetes. Sci. Rep..

[3] Sun Z., Yang D., Tang Z., Ng D.S., Cheung C.Y. (2021). Optical coherence tomography angiography in diabetic retinopathy: An updated review. Eye.

[4] Ogurtsova K., da Rocha Fernandes J.D., Huang Y., Linnenkamp U., Guariguata L., Cho N.H., Cavan D., Shaw J.E., Makaroff L.E. (2017). IDF Diabetes Atlas: Global estimates for the prevalence of diabetes for 2015 and 2040. Diabetes Res. Clin. Pract..

[5] Waheed N.K., Rosen R.B., Jia Y., Munk M.R., Huang D., Fawzi A., Chong V., Nguyen Q.D., Sepah Y., Pearce E. (2023). Optical coherence tomography angiography in diabetic retinopathy. Prog. Retin. Eye Res..

[6] Lechner J., O’Leary O.E., Stitt A.W. (2017). The pathology associated with diabetic retinopathy. Vis. Res..

[7] Miller K., Fortun J.A. (2018). Diabetic Macular Edema: Current Understanding, Pharmacologic Treatment Options, and Developing Therapies. Asia Pac. J. Ophthalmol..

[8] Sim D.A., Keane P.A., Fung S., Karampelas M., Sadda S.R., Fruttiger M., Patel P.J., Tufail A., Egan C.A. (2014). Quantitative analysis of diabetic macular ischemia using optical coherence tomography. Investig. Ophthalmol. Vis. Sci..

[9] Spaide R.F., Fujimoto J.G., Waheed N.K., Sadda S.R., Staurenghi G. (2018). Optical coherence tomography angiography. Prog. Retin. Eye Res..

[10] Spaide R.F. (2015). Optical Coherence Tomography Angiography Signs of Vascular Abnormalization With Antiangiogenic Therapy for Choroidal Neovascularization. Am. J. Ophthalmol..

[11] Yang D., Sun Z., Shi J., Ran A., Tang F., Tang Z., Lok J., Szeto S., Chan J., Yip F. (2022). A Multitask Deep-Learning System for Assessment of Diabetic Macular Ischemia on Optical Coherence Tomography Angiography Images. Retina.

[12] Spaide R.F., Klancnik J.M., Jr Cooney M.J. (2015). Retinal vascular layers imaged by fluorescein angiography and optical coherence tomography angiography. JAMA Ophthalmol..

[13] Weinhaus R.S., Burke J.M., Delori F.C., Snodderly D.M. (1995). Comparison of fluorescein angiography with microvascular anatomy of macaque retinas. Exp. Eye Res..

[14] Mendis K.R., Balaratnasingam C., Yu P., Barry C.J., McAllister I.L., Cringle S.J., Yu D.Y. (2010). Correlation of histologic and clinical images to determine the diagnostic value of fluorescein angiography for studying retinal capillary detail. Investig. Ophthalmol. Vis. Sci..

[15] Hwang T.S., Jia Y., Gao S.S., Bailey S.T., Lauer A.K., Flaxel C.J., Wilson D.J., Huang D. (2015). Optical coherence tomography angiography features of diabetic retinopathy. Retina.

[16] Rosen R.B., Andrade Romo J.S., Krawitz B.D., Mo S., Fawzi A.A., Linderman R.E., Carroll J., Pinhas A., Chui T.Y.P. (2019). Earliest Evidence of Preclinical Diabetic Retinopathy Revealed Using Optical Coherence Tomography Angiography Perfused Capillary Density. Am. J. Ophthalmol..

[17] Schottenhamml J., Moult E.M., Ploner S., Lee B., Novais E.A., Cole E., Dang S., Lu C.D., Husvogt L., Waheed N.K. (2016). An automatic, intercapillary area-based algorithm for quantifying diabetes-related capillary dropout using optical coherence tomography angiography. Retina.

[18] Yasin Alibhai A., Moult E.M., Shahzad R., Rebhun C.B., Moreira-Neto C., McGowan M., Lee D., Lee B., Baumal C.R., Witkin A.J. (2018). Quantifying Microvascular Changes Using OCT Angiography in Diabetic Eyes without Clinical Evidence of Retinopathy. Ophthalmol. Retina.

[19] de Carlo T.E., Bonini Filho M.A., Baumal C.R., Reichel E., Rogers A., Witkin A.J., Duker J.S., Waheed N.K. (2016). Evaluation of Preretinal Neovascularization in Proliferative Diabetic Retinopathy Using Optical Coherence Tomography Angiography. Ophthalmic Surg. Lasers Imaging Retin..

[20] Hwang T.S., Hagag A.M., Wang J., Zhang M., Smith A., Wilson D.J., Huang D., Jia Y. (2018). Automated Quantification of Nonperfusion Areas in 3 Vascular Plexuses With Optical Coherence Tomography Angiography in Eyes of Patients With Diabetes. JAMA Ophthalmol..

[21] Salz D.A., de Carlo T.E., Adhi M., Moult E., Choi W., Baumal C.R., Witkin A.J., Duker J.S., Fujimoto J.G., Waheed N.K. (2016). Select Features of Diabetic Retinopathy on Swept-Source Optical Coherence Tomographic Angiography Compared With Fluorescein Angiography and Normal Eyes. JAMA Ophthalmol..

[22] Cheung C.M.G., Pearce E., Fenner B., Sen P., Chong V., Sivaprasad S. (2021). Looking Ahead: Visual and Anatomical Endpoints in Future Trials of Diabetic Macular Ischemia. Ophthalmologica.

[23] Cheung C.M.G., Fawzi A., Teo K.Y., Fukuyama H., Sen S., Tsai W.S., Sivaprasad S. (2022). Diabetic macular ischaemia—A new therapeutic target?. Prog. Retin. Eye Res..

[24] Bresnick G.H., Condit R., Syrjala S., Palta M., Groo A., Korth K. (1984). Abnormalities of the foveal avascular zone in diabetic retinopathy. Arch. Ophthalmol..

[25] Balaratnasingam C., Inoue M., Ahn S., McCann J., Dhrami-Gavazi E., Yannuzzi L.A., Freund K.B. (2016). Visual Acuity Is Correlated with the Area of the Foveal Avascular Zone in Diabetic Retinopathy and Retinal Vein Occlusion. Ophthalmology.

[26] Safi H., Anvari P., Naseri D., Shenazandi H., Kazemi P., Farsi P., Jafari S., Sedaghat A., Yaseri M., Ghasemi Falavarjani K. (2020). Quantitative analysis of optical coherence tomography angiography metrics in diabetic retinopathy. Ther. Adv. Ophthalmol..

[27] Krawitz B.D., Phillips E., Bavier R.D., Mo S., Carroll J., Rosen R.B., Chui T.Y.P. (2018). Parafoveal Nonperfusion Analysis in Diabetic Retinopathy Using Optical Coherence Tomography Angiography. Transl. Vis. Sci. Technol..

[28] Sim D.A., Keane P.A., Zarranz-Ventura J., Fung S., Powner M.B., Platteau E., Bunce C.V., Fruttiger M., Patel P.J., Tufail A. (2013). The effects of macular ischemia on visual acuity in diabetic retinopathy. Investig. Ophthalmol. Vis. Sci..

[29] Custo Greig E., Brigell M., Cao F., Levine E.S., Peters K., Moult E.M., Fujimoto J.G., Waheed N.K. (2020). Macular and Peripapillary Optical Coherence Tomography Angiography Metrics Predict Progression in Diabetic Retinopathy: A Sub-analysis of TIME-2b Study Data. Am. J. Ophthalmol..

[30] Han R., Gong R., Liu W., Xu G. (2022). Optical coherence tomography angiography metrics in different stages of diabetic macular edema. Eye Vis..

[31] Okumichi H., Itakura K., Yuasa Y., Fukuto A., Kiuchi Y. (2021). Foveal structure in nanophthalmos and visual acuity. Int. Ophthalmol..

[32] Samara W.A., Say E.A., Khoo C.T., Higgins T.P., Magrath G., Ferenczy S., Shields C.L. (2015). Correlation of foveal avascular zone size with foveal morphology in normal eyes using optical coherence tomography angiography. Retina.

[33] Shiihara H., Terasaki H., Sonoda S., Kakiuchi N., Shinohara Y., Tomita M., Sakamoto T. (2018). Objective evaluation of size and shape of superficial foveal avascular zone in normal subjects by optical coherence tomography angiography. Sci. Rep..

[34] Krawitz B.D., Mo S., Geyman L.S., Agemy S.A., Scripsema N.K., Garcia P.M., Chui T.Y.P., Rosen R.B. (2017). Acircularity index and axis ratio of the foveal avascular zone in diabetic eyes and healthy controls measured by optical coherence tomography angiography. Vis. Res..

[35] You Q., Freeman W.R., Weinreb R.N., Zangwill L., Manalastas P.I.C., Saunders L.J., Nudleman E. (2017). Reproducibility of vessel density measurement with optical coherence tomography angiography in eyes with and without retinopathy. Retina.

[36] Chu Z., Lin J., Gao C., Xin C., Zhang Q., Chen C.L., Roisman L., Gregori G., Rosenfeld P.J., Wang R.K. (2016). Quantitative assessment of the retinal microvasculature using optical coherence tomography angiography. J. Biomed. Opt..

[37] Hirano T., Kitahara J., Toriyama Y., Kasamatsu H., Murata T., Sadda S. (2019). Quantifying vascular density and morphology using different swept-source optical coherence tomography angiographic scan patterns in diabetic retinopathy. Br. J. Ophthalmol..

[38] Al-Sheikh M., Falavarjani K.G., Tepelus T.C., Sadda S.R. (2017). Quantitative Comparison of Swept-Source and Spectral-Domain OCT Angiography in Healthy Eyes. Ophthalmic Surg. Lasers Imaging Retina.

[39] Szewczuk A., Zaleska-Żmijewska A., Dziedziak J., Szaflik J.P. (2023). Clinical Application of Adaptive Optics Imaging in Diagnosis, Management, and Monitoring of Ophthalmological Diseases: A Narrative Review. Med. Sci. Monit..

[40] Akyol E., Hagag A.M., Sivaprasad S., Lotery A.J. (2021). Adaptive optics: Principles and applications in ophthalmology. Eye.

[41] Zaleska-Żmijewska A., Wawrzyniak Z.M., Dąbrowska A., Szaflik J.P. (2019). Adaptive Optics (rtx1) High-Resolution Imaging of Photoreceptors and Retinal Arteries in Patients with Diabetic Retinopathy. J. Diabetes Res..

[42] Bakker E., Dikland F.A., van Bakel R., Andrade De Jesus D., Sánchez Brea L., Klein S., van Walsum T., Rossant F., Farías D.C., Grieve K. (2022). Adaptive optics ophthalmoscopy: A systematic review of vascular biomarkers. Surv. Ophthalmol..

[43] Luo T., Gast T.J., Vermeer T.J., Burns S.A. (2017). Retinal Vascular Branching in Healthy and Diabetic Subjects. Investig. Ophthalmol. Vis. Sci..

[44] Ueno Y., Iwase T., Goto K., Tomita R., Ra E., Yamamoto K., Terasaki H. (2021). Association of changes of retinal vessels diameter with ocular blood flow in eyes with diabetic retinopathy. Sci. Rep..

[45] Untracht G.R., Durkee M.S., Zhao M., Kwok-Cheung Lam A., Sikorski B.L., Sarunic M.V., Andersen P.E., Sampson D.D., Chen F.K., Sampson D.M. (2024). Towards standardising retinal OCT angiography image analysis with open-source toolbox OCTAVA. Sci. Rep..

[46] Mehta N., Braun P.X., Gendelman I., Alibhai A.Y., Arya M., Duker J.S., Waheed N.K. (2020). Repeatability of binarization thresholding methods for optical coherence tomography angiography image quantification. Sci. Rep..

[47] Couturier A., Rey P.A., Erginay A., Lavia C., Bonnin S., Dupas B., Gaudric A., Tadayoni R. (2019). Widefield OCT-angiography and fluorescein angiography assessments of nonperfusion in diabetic retinopathy and edema treated with anti-vascularendothelial growth factor. Ophthal-Mology.

[48] Kim Y.J., Yeo J.H., Son G., Kang H., Sung Y.S., Lee J.Y., Kim J.G., Yoon Y.H. (2020). Efficacy of intravitreal Aflibercept injection for Improvement of retinal Nonperfusion in diabetic retinopathy (AFFINITY study). BMJ Open Diabetes Res. Care.

[49] Massengill M.T., Cubillos S., Sheth N., Sethi A., Lim J.I. (2024). Response of Diabetic Macular Edema to Anti-VEGF Medications Correlates with Improvement in Macular Vessel Architecture Measured with OCT Angiography. Ophthalmol. Sci..

[50] Abdelhalim A.S., Abdelkader M., Mahmoud M.S.E., Mohamed Mohamed A.A. (2022). Macular vessel density before and after panretinal photocoagulation in patients with proliferative diabetic retinopathy. Int. J. Retina Vitreous.

[51] Chatziralli I., Dimitriou E., Agapitou C., Kazantzis D., Kapsis P., Morogiannis N., Kandarakis S., Theodossiadis G., Theodossiadis P. (2023). Optical Coherence Tomography Angiography Changes in Macular Area in Patients with Proliferative Diabetic Retinopathy Treated with Panretinal Photocoagulation. Biomedicines.

[52] Fawzi A.A., Fayed A.E., Linsenmeier R.A., Gao J., Yu F. (2019). Improved Macular Capillary Flow on Optical Coherence Tomography Angiography After Panretinal Photocoagulation for Proliferative Diabetic Retinopathy. Am. J. Ophthalmol..

[53] Takahashi A., Nagaoka T., Sato E., Yoshida A. (2008). Effect of panretinal photocoagulation on choroidal circulation in the foveal region in patients with severe diabetic retinopathy. Br. J. Ophthalmol..

[54] Zhao T., Chen Y., Liu D., Stewart J.M. (2020). Optical Coherence Tomography Angiography Assessment of Macular Choriocapillaris and Choroid Following Panretinal Photocoagulation in a Diverse Population with Advanced Diabetic Retinopathy. Asia Pac. J. Ophthalmol..

[55] Lorusso M., Milano V., Nikolopoulou E., Ferrari L.M., Cicinelli M.V., Querques G., Ferrari T.M. (2019). Panretinal Photocoagulation Does Not Change Macular Perfusion in Eyes with Proliferative Diabetic Retinopathy. Ophthalmic Surg. Lasers Imaging Retin..

[56] Stefánsson E. (2009). Physiology of vitreous surgery. Graefes Arch. Clin. Exp. Ophthalmol..

[57] Petrou P., Angelidis C.D., Andreanos K., Kanakis M., Kandarakis S., Karamaounas A., Papakonstantinou E., Mamas N., Droutsas K., Georgalas I. (2021). Reduction of Foveal Avascular Zone After Vitrectomy Demonstrated by Optical Coherence Tomography Angiography. Cureus.

[58] Russell J.F., Scott N.L., Townsend J.H., Shi Y., Gregori G., Crane A.M., Flynn H.W., Sridhar J., Rosenfeld P.J. (2021). Wide-Field Swept-Source Optical Coherence Tomography Angiography of Diabetic Tractional Retinal Detachments before and after Surgical Repair. Retina.

